# Fenofibrate Augments the Sensitivity of Drug-Resistant Prostate Cancer Cells to Docetaxel

**DOI:** 10.3390/cancers11010077

**Published:** 2019-01-11

**Authors:** Marcin Luty, Katarzyna Piwowarczyk, Anna Łabędź-Masłowska, Tomasz Wróbel, Małgorzata Szczygieł, Jessica Catapano, Grażyna Drabik, Damian Ryszawy, Sylwia Kędracka-Krok, Zbigniew Madeja, Maciej Siedlar, Martyna Elas, Jarosław Czyż

**Affiliations:** 1 Department of Cell Biology, Faculty of Biochemistry, Biophysics and Biotechnology, Jagiellonian University, Gronostajowa 7, 30-387 Kraków, Poland; marcin.luty@uj.edu.pl (M.L.); katarzyna.szpak@uj.edu.pl (K.P.); anna.labedz-maslowska@uj.edu.pl (A.L.-M.); 1tomaszwrobel@gmail.com (T.W.); 1catapanojessica@gmail.com (J.C.); damian.ryszawy@uj.edu.pl (D.R.); z.madeja@uj.edu.pl (Z.M.); 2 Department of Biophysics, Faculty of Biochemistry, Biophysics and Biotechnology, Jagiellonian University, Gronostajowa 7, 30-387 Kraków, Poland; gosia.szczygiel@uj.edu.pl (M.S.); martyna.elas@uj.edu.pl (M.E.); 3 Department of Transplantology, Institute of Paediatrics, Faculty of Medicine, Jagiellonian University Medical College, 265 Wielicka Str., 30-663 Kraków, Poland; gd22@interia.pl; 4 Department of Physical Biochemistry, Faculty of Biochemistry, Biophysics and Biotechnology, Jagiellonian University, Gronostajowa 7, 30-387 Kraków; and Proteomics and Mass Spectrometry Laboratory, Malopolska Centre of Biotechnology, Jagiellonian University, Gronostajowa 7A, 30-387 Krakow, Poland; sylwia.kedracka-krok@uj.edu.pl; 5 Department of Clinical Immunology, Institute of Paediatrics, Faculty of Medicine, Jagiellonian University Medical College, 265 Wielicka Str., 30-663 Kraków, Poland; misiedla@cyf-kr.edu.pl

**Keywords:** fenofibrate, chemotherapy, prostate cancer, drug-resistance, cancer microevolution

## Abstract

Metronomic agents reduce the effective doses and adverse effects of cytostatics in cancer chemotherapy. Therefore, they can enhance the treatment efficiency of drug-resistant cancers. Cytostatic and anti-angiogenic effects of fenofibrate (FF) suggest that it can be used for the metronomic chemotherapy of drug-resistant prostate tumors. To estimate the effect of FF on the drug-resistance of prostate cancer cells, we compared the reactions of naïve and drug-resistant cells to the combined treatment with docetaxel (DCX)/mitoxantrone (MTX) and FF. FF sensitized drug-resistant DU145 and PC3 cells to DCX and MTX, as illustrated by their reduced viability and invasive potential observed in the presence of DCX/MTX and FF. The synergy of the cytostatic activities of both agents was accompanied by the inactivation of P-gp-dependent efflux, dysfunction of the microtubular system, and induction of polyploidy in DCX-resistant cells. Chemical inhibition of PPARα- and reactive oxygen species (ROS)-dependent pathways by GW6471 and N-acetyl-L-cysteine, respectively, had no effect on cell sensitivity to combined DCX/FF treatment. Instead, we observed the signs of adenosine triphosphate (ATP) deficit and autophagy in DCX/FF-treated drug-resistant cells. Furthermore, the cells that had been permanently propagated under DCX- and DCX/FF-induced stress did not acquire DCX/FF-resistance. Instead, relatively slow proliferation of DCX-resistant cells was efficiently inhibited by FF. Collectively, our observations show that FF reduces the effective doses of DCX by interfering with the drug resistance and energy metabolism of prostate cancer cells. Concomitantly, it impairs the chemotherapy-induced microevolution and expansion of DCX/FF-resistant cells. Therefore, FF can be applied as a metronomic agent to enhance the efficiency of palliative chemotherapy of prostate cancer.

## 1. Introduction

Versatile self-defense/protective systems are functional in prokaryotic and eukaryotic cells. They reduce cellular susceptibility to adverse environmental conditions and to chemical pathogens. In metazoa, multi-drug resistance (MDR) systems preserve the tissue and organ homeostasis by mediating the efflux of chemical pathogens, enhancing the drug metabolism and DNA repair, and elevating autophagy in intoxicated cells [1]. Impaired MDR negatively affects the cells’ microenvironmental adaptability but the over-activity of MDR pathways can interfere with the cytostatic activity of chemotherapeutics. For instance, the activation of self-defense systems in bacteria and fungi reduces the efficiency of antibiotic therapies, whereas the efficiency of cancer chemotherapy is impaired by over-active MDR systems in cancer cells [2]. The activity of membrane proteins that pump chemicals out of the cells and reduce the intracellular levels of chemotherapeutic agents is perhaps the most prominent MDR system in cancer cells; however, the activity of other self-defense systems is also augmented in cancer cells. For instance, DNA repair systems and autophagy of damaged organelles additionally augments the drug-resistance of cancer cells. Along with the formation of invasive cancer cell sub-populations, these events collectively lead to the ultimate recurrence of malignant tumors after temporary cancer remission [3]. Thus, drug-resistance of cancer cells remains a major challenge in current clinical oncology [1]. 

Prolonged administration of chemotherapeutic drugs promotes the “survival of the fittest” cells [4], leading to the selective intratumoral microevolution/expansion of drug-resistant cell lineages. Their expansion in primary tumors eventually results in tumor recurrence after the cessation of chemotherapy. Functional links exist between multi-drug resistance, multipotency and malignancy of cancer cells. For instance, cancer stem cells (CSCs; [5,6]) are characterized by high activity of drug-efflux systems [7,8], which facilitates their survival upon exposition to the long-term chemotherapeutic stress, and underlies subsequent tumor recurrence and metastasis [9,10]. These systems are also over-active in cancer cells that have undergone epithelial-mesenchymal transition (EMT). This leads to enhanced drug-resistance of the cells that constitute the invasive front(s) of tumors [11,12,13], including prostate cancer, which is a leading cause of cancer-related deaths in Western countries. So far, ca. 50 MDR proteins has been identified [7]. Over-activation of at least 3 proteins (ABCB1, ABCC1 i ABCG2) was observed in post-EMT prostate cancer cells [14]. The interrelations between the malignancy and the activity of MDR systems in prostate cancer cells enforce the gradual increase of chemotherapeutic doses against continuously more aggressive tumor cells that reside within the weakening organism of the patient. The dreadful effects of this vicious cycle are especially significant in palliative treatment of older patients, who are over-sensitive to the adverse effects of chemotherapeutics. These effects enforce premature cessations of treatment, which facilitate the expansion of invasive drug-resistant cells, and prostate tumor recurrence and progression.

The way out of this dilemma is provided by metronomic agents that interfere with the drug-resistance and malignancy of cancer cells, thus reducing the effective doses of chemotherapeutics [15]. Recently, the application of fenofibrate (FF; propan-2-yl 2-{4-[(4-chlorophenyl) carbonyl]phenoxy}-2-methylpropanoate) has been suggested for cancer treatment [16,17] including the palliative strategies of prostate chemotherapy [18,19]. FF is a well-tolerated, Food and Drug Administration (FDA)-approved anti-hyperlipidemic and vasoactive drug, which lowers serum levels of triglycerides and cholesterol, and improves the low-density lipoprotein:high-density lipoprotein (LDL:HDL) ratio [20]. PPARα-dependent and PPARα-independent signaling is involved in cancer cell reactions to FF; however, the interference of FF with the chemoresistance of cancer cells has not yet been analyzed. We hypothesized that FF can increase the sensitivity of prostate cancer cells to chemotherapeutics, thus increasing their efficiency and attenuating systemic adverse effects of chemotherapy. To verify this notion, we analyzed the synergy of cytostatic and anti-invasive effects of docetaxel (DCX), mitoxantrone (MTX) and FF on drug-resistant prostate cancer cells. Furthermore, we evaluated the interference of FF with their multi-drug resistance systems. Finally, we focused on self-protective mechanisms, which are activated by prostate cancer cells in response to combined DCX/FF treatment. Our data show that FF can reduce the effective doses of DCX in the therapy of prostate cancer.

## 2. Results

### 2.1. Fenofibrate (FF) Increases the Sensitivity of DU145 Cells to Docetaxel (DCX) 

**Figure 1:**
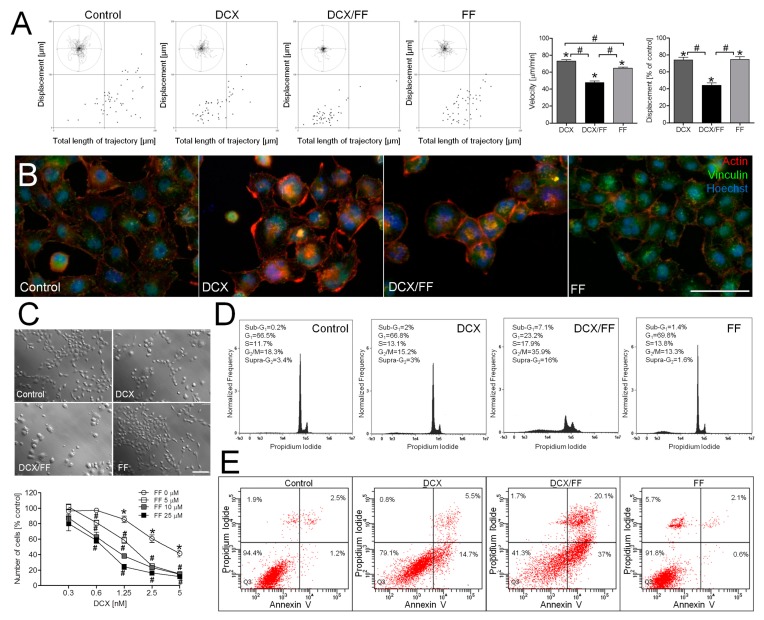
Additive effects of fenofibrate (FF) and docetaxel (DCX) on the viability and invasive potential of naïve DU145 cells. (**A**) Naïve DU145 cells were incubated in the presence of DCX (2.5 nM) and/or FF (25 μM) and their motility was estimated immediately after drug administration. Cell trajectories are depicted as circular diagrams (axis scale in µm) drawn with the initial point of each trajectory placed at the origin of the plot (registered for 6 h; N > 50). Dot-plots and column charts show movement parameters at the single cell and population level, respectively (plotted as % of control). (**B**) Intracellular localization of actin (red) and vinculin (green) were visualized by immunofluorescence in DU145 cells incubated in the presence of DCX/FF (2.5 nM/25 μM) for 48 h. (**C**) DU145 cells were cultivated in the presence of DCX (0.3–5 nM) and/or FF (5–25 μM). Their morphology (upper panel; 2.5 nM DCX/25 μM FF) and proliferation (lower panel; calculated as % of control) were estimated after 48 h. (**D**,**E**) The effect of the long-term DCX/FF (2.5 nM/25 μM) treatment on the viability of DU145 cells was estimated with fluorescence-activated cell sorting (FACS)-assisted DNA content (after 48 h; **D**) and annexinV/PI assay (after 72 h; **E**). DNA histograms and compensated dot-plots comprise 30,000/50,000 events, classified based on their bright field ratios and/or nuclear contrast. Statistical significance was analyzed with the non-parametric Mann-Whitney (**A**; vs. non-treated control (* *p* ≤ 0.05) or vs. controls indicated by the backets; **#** *p* ≤ 0.05); or by t-Student test (**C**; vs. non-treated control (* *p* ≤ 0.05) or vs. DCX-treated variant (0 μM FF; **#** *p* ≤ 0.05). Error bars represent standard error of the mean (SEM). Scale bar: 50 µm (**B**) and 100 µm (**C**). Data are representative of at least three independent experiments (N > 3). Note that FF increases the sensitivity of DU145 cells to DCX.

The additive inhibitory effects of combined docetaxel (DCX)/fenofibrate (FF) treatment on the welfare of prostate cancer cells are illustrated by the inhibition of naïve DU145 cell motility observed immediately after the administration of both agents (Figure 1A). A less-pronounced inhibition of DU145 cell motility and displacement was observed, when DCX and FF were administered separately; however FF efficiently attenuated gap junctional intercellular communication (GJIC) in DU145 populations after 24 h-long treatment (Figure S1A). These effects were followed by prominent actin cytoskeleton rearrangements in DCX- and DCX/FF-treated cells (Figure 1B) and correlated with a dose-dependent inhibitory effect of DCX and FF on DU145 proliferation (Figure 1C, cf. Figure S1B). 

A distinct inhibition of DU145 proliferation was observed when DCX/FF was administered at the concentration between 1.25 nM/5 μM. Additive effects of DCX/FF on cell motility and proliferation were also observed in the populations of human prostate cancer PC3 cells (Figure S2A–D). Furthermore, DNA content analyses revealed the induction of apoptosis and polyploidy in DCX/FF-treated DU145 populations, as illustrated by the abundance of their sub-G_1_/supra-G_2_ fractions, respectively (Figure 1D). The apoptotic response of DU145 cells to the combined DCX/FF treatment was further confirmed by AnnexinV/PI assay that showed a prominent fraction of annexinV^+^ cells after DCX/FF administration in the absence of a distinct pro-apoptotic activity of separately administered agents (Figure 1E). Collectively, these data show that FF increases the sensitivity of prostate cancer cells to DCX.

### 2.2. FF Interferes with DCX-Resistance of Prostate Cancer Cells 

To estimate the interference of FF with the drug-resistance of prostate cancer cells, we have established 2 sub-lines of DCX-resistant DU145 cells (Figure S3; see Section 4 Materials and Methods) by exposing naïve DU145 cells to increasing doses of DCX. 

**Figure 2:**
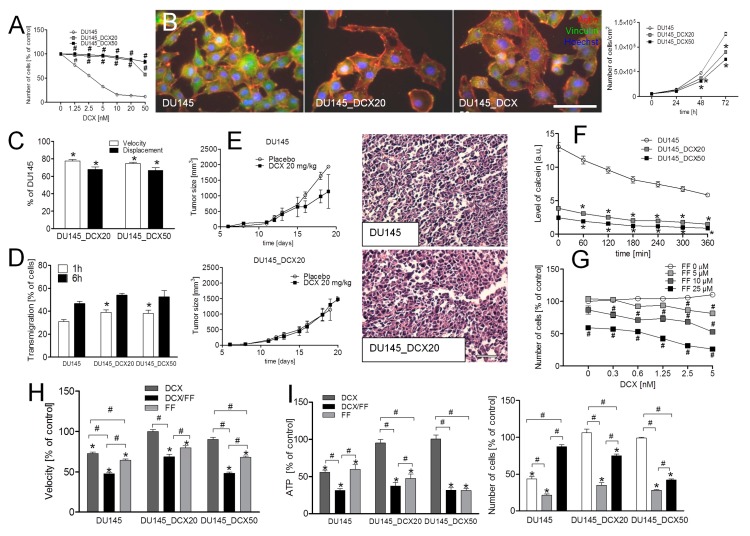
FF interferes with the DCX-resistance of DU145 cells. (**A**) Naïve DU145 and DCX-resistant DU145 cells (DU145_DCX20 and DU145_DCX50; cf. Figure S3 in Supplementary material) were cultivated in the presence of DCX (1.25–50 nM) and their proliferation was estimated after 48 h. (**B**) Comparison of the morphology, actin cytoskeleton architecture (left) and proliferation (right) of DU145, DU145_DCX20 and DU145_DCX50 cells in control conditions. (**C**,**D**) Motility (C) and trans-endothelial migration efficiency of DU145 and DCX-resistant DU145 cells (D) was estimated in actin/vinculin-stained specimens. DU145 cells were additionally stained with CellTracer Orange CMRA. Transmigration values show a percent of cancer cells that penetrated the adjacent endothelial barrier. (**E**) DU145 and DU145_DCX20 cells were subcutaneously injected into severe combined immunodeficient (SCID) mice (1.5 × 10^5^) and the growth of tumors was estimated in the absence/presence of DCX (20 mg/kg b.w). Images show the structure of tumors visualized by hematoxilin/eosin staining. (**F**) The activity of efflux pumps in DCX-resistant DU145 cells was estimated with calcein efflux assay in control conditions (plotted as fluorescence intensity (a.u.)). (**G**) DU145_DCX20 cells were cultivated in the presence of DCX (0.3–5 nM) and/or FF (5–25 μM). Their proliferation was estimated after 48 h (plotted as % of control). (**H**,**I**) Naïve and DCX-resistant DU145 cells were treated with 2.5 nM DCX/25 μM FF and their motility was estimated with time-lapse videomicroscopy immediately afterwards (H) or (I) their viability (ATP levels (left)/proliferation (right)) were estimated after 48 h (calculated as % of control). Data representative of at least three independent experiments (N > 3). Statistical significance was analyzed with the t-Student (**A**,**B**,**D**–**G**,**I**) or with the non-parametric Mann-Whitney test (**C**,**H**) vs. non-treated control (* *p* ≤ 0.05), vs. DCX-treated variant (**#** *p* ≤ 0.05; **A**) or vs. the variant indicated by the brackets (**#** *p* ≤ 0.05). Error bars represent SEM. Scale bar: 50 µm. Note the additive inhibitory effects of FF and DCX on the welfare and motility of DCX-resistant DU145 cells.

Drug-resistance of DU145_DCX20 and DU145_DCX50 cells was manifested by negligible effects of DCX (Figure 2A) and MTX on their proliferation (Figure S4A). DU145_DCX50 cells, which were pre-selected in the presence of higher DCX concentrations, were slightly more resistant to both agents than DU145_DCX20 cells (Figure 2A; cf. Figure S4A). Both drug-resistant cell lines displayed epithelioid phenotype with prominent focal contacts, relatively low proliferation rate (Figure 2B) and Cx43^+^ gap junctions (Figure S4B). They were also characterized by a slightly less efficient motility than DU145 cells (Figure 2C), but relatively high transmigration potential in vitro (Figure 2D; cf. Figure S4C). In comparison to DU145 tumors, DU145_DCX20 tumors grew relatively slowly in control in vivo conditions, but were considerably less vulnerable to DCX stress (Figure 2E). DCX-resistance of DU145_DCX20/50 cells correlated with the high efficiency of efflux systems (ABC transporters) in these cells, illustrated by a high calcein efflux assay (Figure 2F; cf. Figure S4D). Accordingly, DCX did not affect their residual GJIC (Figure S4E) and motility in vitro (Figure S5A). FF increased the susceptibility of DU145_DCX20 cells to DCX (Figure 2G and Figure S5B) and to MTX (Figure S4A) in a dose-dependent manner. This effect was also manifested by the inhibition of cell motility in DCX/FF-treated populations (Figure 2H, cf. Figure S5A) and by the additive cytostatic effect of both agents on the viability of drug-resistant cells. This is illustrated by their decreased viability (measured by adenosine triphosphate (ATP) levels at the population level) and prolonged doubling times in the presence of 2.5 nM DCX/25 μM FF (Figure 2I, cf. Figure S5C–E). Notably, DCX/FF also exerted additive cytostatic effects on drug-resistant PC3 cells, which confirms biological significance of this phenomenon (cf. Figure S2F–H). These observations show that FF augments the sensitivity of drug-resistant prostate cancer cells to the cytostatic activity of DCX. 

### 2.3. FF Attenuates the Invasive Potential of DCX-Resistant DU145 Cells 

**Figure 3:**
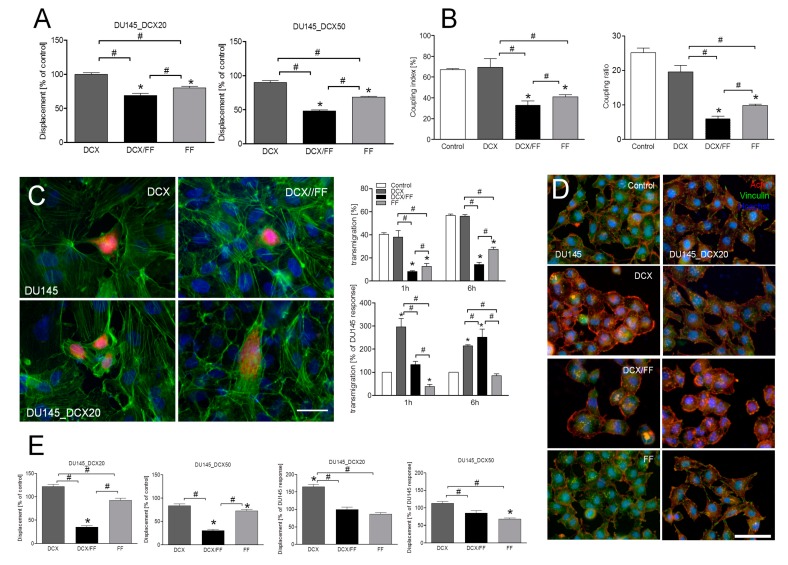
Combined DCX/FF treatment interferes with the invasive behavior of DCX-resistant DU145 cells. (**A**) Displacement of DU145 and DCX-resistant DU145 cells was estimated with time-lapse videomicroscopy immediately after the administration of DCX (2.5 nM) and/or FF (25 μM). Column bars show averaged cell displacement (registered for 6 h; N > 50). (**B**) DCX-resistant DU145 cells were incubated in the presence of 2.5 nM DCX and/or 25 μM FF for 24 h. GJIC was estimated by calcein transfer assay and calculated as coupling index (left panel) and coupling ratio (right panel). (**C**) DU145 and DU145_DCX20 cells were cultivated as in (**B**) and their transmigration through HUVEC continuum was estimated by transendothelial migration assay. Transmigration values show the percent of DU145 cells that disrupted/penetrated endothelial layer (upper panel) or the percent of penetrating DU145_DCX20 cells calculated in relation to the transmigation of naïve DU145 cells in the corresponding conditions (lower panel). (**D,E**) Intracellular distribution of actin/vinculin was visualized in DU145 and DCX-resistant DU145 cells incubated in the presence of 2.5 nM DCX/25 μM FF for 48 h with fluorescence microscopy (**D**) or cell displacement was estimated with time-lapse videomicroscopy 48 h after the administration of the drugs (**E**). Column charts show movement parameters at the single cell and population level, respectively. Column bars show averaged cell displacement of the cells cultivated in different conditions (left, measured as % of control), or calculated in relation to DU145 cell movement (right). Data representative of at least three independent experiments (N > 3). Error bars represent SEM. Statistical significance was analyzed with the non-parametric Mann–Whitney test (**A**,**E**) or t-Student test (**B**,**C**) vs. non-treated control (* *p* ≤ 0.05) or vs. the variant indicated by the brackets (**#** *p* ≤ 0.05). Scale bars: 50 µm. Note that FF strengthens the long-term effect of DCX on the invasive potential of DCX-resistant DU145 cells.

Further analyses of the invasive behavior of DCX/FF-treated of prostate cancer cells enabled us to address the therapeutic significance of the inhibitory FF effect on their DCX-resistance. Not surprisingly, 2.5 nM DCX had little short-term effect on the displacement of DCX-resistant cells (Figure 3A; cf. Figure S5A). This is illustrated by relatively high values of this parameter in DCX-treated DU145_DCX20/50 populations. DCX also exerted minute effects on GJIC in DU145_DCX20 populations, whereas FF treatment considerably impaired this parameter, both in the absence and the presence of DCX (Figure 3B; cf. Figure S6A). Transendothelial penetration assays demonstrated that the inhibitory effect of DCX on the efficiency of transendothelial migration of DCX-resistant DU145 cells was augmented by FF (Figure 3C; cf. Figure S6B). DCX-resistant cells were even more sensitive to FF treatment than DU145 cells. It also affected polarization of both naïve and DCX-resistant cell shapes (Figure 3D; cf. Figure S6C) and interfered with their long-term displacement (Figure 3E; cf. Figure S6D,E). Long-term interference of DCX/FF with actin cytoskeleton architecture and with displacement of DU145_DCX20/DCX50 cells may account for the impaired transmigration of DCX-resistant cells. When extrapolated to in vivo situation, these data indicate that FF can interfere with prostate cancer invasion and systemic dissipation. 

### 2.4. FF Inhibits the Activity of Drug-Efflux Pumps in DU145 Cells 

**Figure 4:**
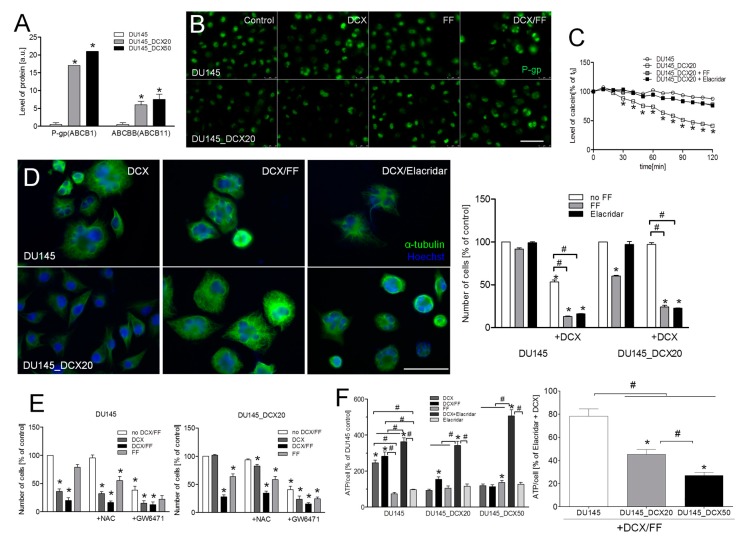
FF interferes with the activity of multi-drug resistance (MDR) transporters in DCX-resistant DU145 cells. (**A**) Abundance of the peptides identified by shotgun tandem mass spectrometry corresponding to of MDR protein levels in DU145 and DCX-resistant cells. MDR protein levels in DU145 and their DCX-resistant counterparts cells were identified according to semi-quantitative analysis of peptide-spectrum matches (PSMs) obtained in liquid chromatography-mass spectrometry LC–MS/MS measurements. (**B**) The effect of DCX/FF on the intracellular localization of P-gp was visualized with fluorescence microscopy in naïve DU145 and DU145_DCX20 cells 48 h after the administration of 2.5 nM DCX/25 μM FF. (**C**) Naïve DU145 and DU145_DCX20 cells were incubated in the presence of elacridar (0.1 μM) or FF (25 μM). The efficiency of their efflux systems was measured by calcein efflux assay. (**D**) The effect of DCX/elacridar/FF on the architecture of microtubules in DU145 and DCX-resistant DU145 cells (left) and on their proliferation (right) was estimated with immunofluorescence and cell counting, respectively. (**E**) Naïve DU145 (left) and DU145_DCX20 cells (right) were incubated in the presence of DCX/FF and GW6471 (10 μM) or N-acetyl-L-cysteine (1 mM; NAC), and their proliferation was estimated after 48 h (as % control). (**F**) The effect of elacridar (0.1 μM) and/or 2.5 nM DCX/25 μM FF on ATP levels was estimated in single DU145 and DCX-resistant DU145 cells after 48 h of incubation and calculated as % of the untreated control (left) and as % of DCX/elacridar-treated cells (right). Data representative of at least three independent experiments (N > 3). Error bars represent SEM. Statistical significance was analyzed with t-Student test vs. non-treated DU145 control, non-treated DU145_DCX20 control (**E**; right; * *p* ≤0.05) or vs. the variant indicated by the brackets (**#** *p* ≤ 0.05). Scale bar: 50 (**D**) or 100 µm (**B**). Note that the DCX-resistance of DU145_DCX20 and DU145_DCX50 cells correlates with relatively high activity of efflux systems in these cells. Inhibition of this system in DCX/FF-treated cells correlates with PPARα/ROS-independent reduction of intracellular ATP levels.

The cytostatic and anti-invasive effects of FF on drug-resistant cells prompted us to estimate FF interference with the activity of their detoxication systems. Tandem mass spectrometry suggested the elevation of P-gp (ABCB1) levels in DCX-resistant DU145 cells (Figure 4A). The expression of P-gp was also revealed in naïve DU145 and DU145_DCX20 cells by immunofluorescence studies in control conditions and in the presence of DCX/FF (Figure 4B). The inhibition of P-gp by elacridar (0.1 μM) attenuated the calcein efflux from DU145_DCX20 cells to the levels characteristic for DU145 cells (Figure 4C; cf. Figure S7A). FF induced the inhibition of dye efflux in drug-resistant cells, which corresponded to that observed in the presence of elacridar (Figure 4C). This effect was accompanied by the disorganization of microtubular systems both in DCX/elacridar- and DCX/FF-treated cells (Figure 4D, left; cf. Figure S7B, C) and the inhibition of their proliferation (Figure 4D; right). 

Notably, the chemical inhibition of ABCC1 (by sulfinpyranoze) and ABCG2 (by fumitremorgin C) had no significant effect on calcein efflux, even though both agents (especially fumitremorgin C) significantly inhibited cell proliferation (Figure S7D). Furthermore, elacridar increased the sensitivity of drug-resistant PC3 cells to DCX (Figure S7E). These observations confirm the dominant role of P-gp in the determination of DU145/PC3 drug-resistance. They also indicate that FF impairs the drug-resistance of DU145_DCX20 cells via the interference with P-gp function. 

To identify the signaling pathways responsible for the interference of FF with efflux systems, we applied DCX and FF to naïve DU145 and DU145_DCX20 cells in the presence of chemical PPARα and reactive oxygen species (ROS) inhibitor (GW6471 and N-acetyl-L-cysteine (NAC), respectively, Figure 4E). The lack of their attenuating effects on the cytostatic activity of DCX/FF indicates that FF interferes with their DCX-resistance in a PPARα/ROS-independent manner. On the other hand, we noticed increased ATP levels in DCX-pretreated DU145_DCX20 cells upon the inhibition of P-gp activity by elacridar (Figure 4F) and a less pronounced ATP accumulation in DCX/FF-treated cells. Collectively, these data indicate that FF impairs energy metabolism that interferes with the activity of P-gp-induced DCX efflux in DCX-resistant DU145 cells. 

### 2.5. Combined DCX/FF Stress Induces Autophagy in DCX-Resistant Cells

**Figure 5:**
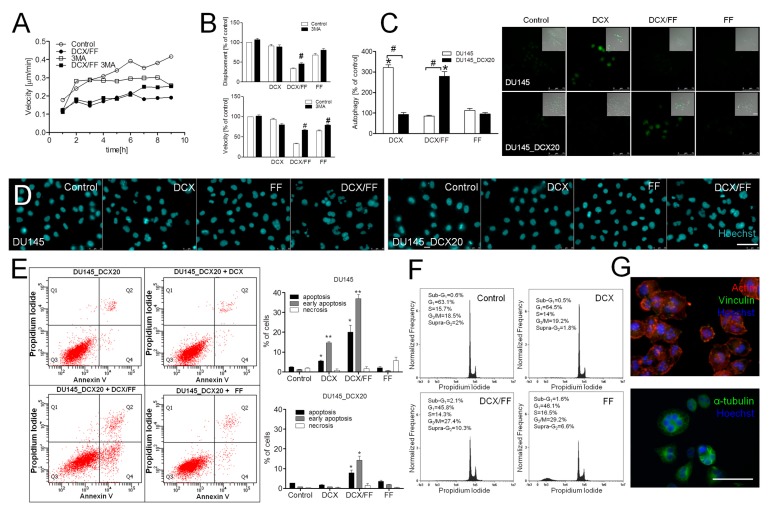
Combined DCX/FF treatment induces autophagy in DCX-resistant DU145. (**A**) Exemplary instantaneous speeds of single DU145_DCX20 cells estimated with time-lapse videomicroscopy (for 9 h) immediately after the administration of DCX and/or FF in the presence/absence of 0.5 μM 3-methyladenine (3MA). (**B**) Averaged motility and displacement of DCX- and DCX/FF-treated DU145_DCX20 cells in the presence/absence of 3MA (plotted as % of control). (**C**) DU145 and DU145_DCX20 cells were subjected to DCX and/or FF for 48 h and the activity of autophagosomes was visualized by autophagy detection kit. (**D**) DU145 and DU145_DCX20 cells were cultivated as in (**C**) and stained with Hoechst33258 to visualize their nuclei by fluorescence microscopy. (**E**) Pro-apoptotic activity of DCX and/or FF was estimated in DU145_DCX20 populations after 72 h with annexinV/PI assay and compared with pro-apoptotic activity of DCX/FF on DU145 cells (right panel). (**F**) DU145_DCX20 cells were cultivated as in (**C**) for 48 h and stained with propidium iodide in the presence of rybonucleaseA. DNA contents were estimated by FACS. (**G**) Morphology of polyploid cells in DCX/FF-treated populations of DU145_DCX20 cells stained against F-actin, vinculin and α-tubulin. Histograms and compensated dot-plots comprise 30,000 and 50,000 events, respectively, classified based on their bright field ratios and nuclear contrast. Error bars represent SEM. Statistical significance was analyzed with t-Student test vs. non-treated DU145 control (**B**; * *p* ≤ 0.05), DCX/FF-treated control (**B**) or with the variant indicated by the brackets (**#** *p* ≤ 0.05). Scale bar: 50 µm. Note DCX/FF-induced autophagy in DU145_DCX20 cells and their relatively low resistance to DCX/FF-induced apoptosis.

Cancer cells activate numerous self-defense systems in response to extrinsic stress. Therefore, we further estimated whether the activation of stress responses can protect drug-resistant prostate cancer cells against combined DCX/FF treatment. Apparently, it activated stress reactions of DCX-resistant cells as illustrated by the increased phosphorylation of AMP-activated kinase (AMPK;not shown). Concomitantly, 3-methyladenine (3MA) induced the short-term retardation of DCX/FF-induced inhibition of DU145_DCX20 motility at the single cell (Figure 5A) and population level (Figure 5B). Because 3MA is an inhibitor of autophagy in energy-depleted cells [21], these observations suggest that DCX/FF induces their “suicidal” autophagy. However, intensified autophagy was also seen in DCX-resistant cells that have undergone long-term (72 h) DCX/FF treatment (Figure 5C). Thus, a small sub-population of DU145_DC20 cells can activate "protective" autophagy in response to FF-induced ATP deficit, which determines cell survival upon DCX/FF treatment. Its protective effect is not strong enough to fully counteract the cytotoxic effects of DCX/FF, as illustrated by disorganization of microtubules (cf. Figure 4) and nuclear distortions (Figure 5D) in DCX/FF-treated DU145_DCX20 cells. On the other hand, a less pronounced apoptosis (Figure 5E) and the induction of polyploidy (Figure 5F,G) in DCX/FF-treated DU145_DCX20 cells (compared to naïve DU145 cells) indicated that long-term DCX treatment might have induced a certain level of DCX/FF-resistance in DU145 cells. 

### 2.6. Long-Term DCX/FF Treatment Does Not Prompt the Microevolution of DCX/FF-Resistant DU145 Cells 

**Figure 6:**
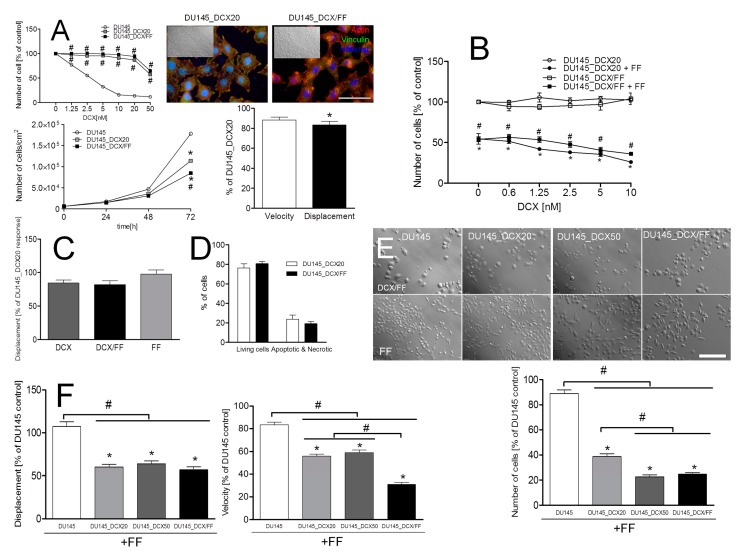
Long-term DCX/FF treatment of prostate cancer cells does not prompt their DCX/FF-resistance. (**A**) DCX-resistance and phenotypic properties of DU145_DCX/FF cells. DU145_DCX/FF cells were cultivated in the presence of DCX (cf. Figure 2A) and their proliferation was compared to the proliferation of DU145_DCX20 cells (upper left). Concomitantly, actin cytoskeleton architecture (upper right), motility (lower right) and proliferation (lower left) was estimated in the absence of chemotherapeutics. (**B**) DU145_DCX/FF cells were incubated in the presence of DCX (0.6-10 nM) and/or FF (25 μM) and their proliferation was estimated after 48 h. (**C**) Motility of DU145_DCX/FF cells cultivated in the presence of DCX (2.5 nM) and/or FF (25 μM) for 48 h, plotted as percent of DU145_DCX20 control. Column charts show movement parameters at the population level. (**D**) Comparison of apoptotic responses of DU145_DCX20 and DU145_DCX/FF cells to the combined DCX/FF treatment (cf. Figure S8). (**E,F**) Sensitivity of DU145, DU145_DCX20./50 and DU145_DCX/FF cells to FF, estimated with proliferation (**E**) and motility tests (**F**). Data representative of at least three independent experiments (N > 3). Statistical significance was analyzed with t-Student test vs. non-treated DU145 control (B; * *p* ≤ 0.05) or DCX-treated DU145 control (**A**), non-treated DU145_DCX/FF control (**B**) and with the variants indicated by the brackets (**#** *p* ≤ 0.05). Error bars represent SEM. Scale bars: 50 (**A**) and 100 µm (**E**). Note the sensitivity of DU145_DCX/FF cells to combined DCX/FF treatment and the retardation of DU145_DCX/FF cell growth in control conditions.

To further elucidate whether the combined DCX/FF treatment can prompt the microevolution of the DCX/FF-resistant cells, we subjected naïve DU145 cells to the long-term DCX/FF exposition protocol (Figure S3; see Section 4 Materials and Methods). This allowed us to establish an epithelioid cell line (DU145_DCX/FF), characterized by the motility, invasive potential in vitro and DCX-resistance of DU145_DCX/FF cells were similar to these of DU145_DCX20 cells (Figure 6A). Interestingly, these cells retained relatively high susceptibility to the combined DCX/FF treatment, as illustrated by their considerably inhibited proliferation (Figure 6B) and the motility similar to that estimated for DU145_DCX20 cells in corresponding conditions (Figure 6C; cf. Figure S8A). Concomitantly, the magnitude of their apoptotic response to combined DCX/FF treatment was similar to that observed in DU145_DCX20 populations (Figure 6D, cf. Figure S8B). Collectively, these observations indicate that long-term exposure of DU145 cells to DCX/FF does not prompt their resistance to combined DCX/FF treatment. 

Instead, we observed a correlation between DCX-resistance of prostate cancer cells and their sensitivity to FF. It is manifested by a much stronger inhibition of their proliferation in the presence of FF (Figure 6E). High sensitivity of DU145_DCX20/50 and DU145_DCX/FF cells to FF treatment is also illustrated by a considerable inhibition of motility in the presence of FF (Figure 6F, cf. Figure S8C). Together with their relatively slow proliferation rate in control conditions, these data demonstrate that DCX-resistance implies metabolic reprogramming of prostate cancer cells that increases their sensitivity to FF, interferes with their propagation in the presence of FF, and impairs the microevolution of “super-resistant” cells.

## 3. Discussion

According to Hanahan and Weinberg, the basic hallmarks of cancer include the replicative immortality of cancer cells, their resistance to growth suppressors and pro-apoptotic signals, and overactive proliferative signaling [22,23]. These features underlie a rapid and tissue-independent expansion of cancer cells and the hyperplasia/hypertrophy of primary tumors. Both processes are further facilitated by reprogramming of energy metabolism and the pro-angiogenic potential of cancer cells. Together with tumor cells’ invasiveness and immunoresistance, these hallmarks enhance the micro-environmental adaptability of cancer cells and their capability of colonizing new “niches”. Till now, the interference of fenofibrate (FF) with the malignancy of tumor cells was predominantly considered in terms of its cytostatic, pro-apoptotic and anti-invasive activity [24,25,26,27,28]. Only a few studies have considered the synergy of cytostatic effects of FF and chemotherapeutic drugs [29,30]. Our study is the first to scrutinize the anti-cancer potential of fenofibrate in the context of the microevolution of drug-resistance in prostate cancers. It shows that the interference of FF with P-gp-dependent efflux systems in drug-resistant prostate cancer cells can increase their sensitivity to chemotherapeutics, including docetaxel and mitoxantrone. Next, we demonstrate that the microevolution of drug-resistance can increase cellular sensitivity to the cytostatic effect of fenofibrate. Both these effects potentially can impair the microevolution and progression of drug-resistant prostate tumors in vivo. Notably, these effects were observed in the presence of <25 μM FF, i.e., within the range of its tolerable serum concentrations (up to 100 μM, [31]). This confirms the potential of FF for application in the palliative treatment of drug-resistant prostate tumors.

Chemotherapy usually leads to a transient syndrome relief, but the selective pressure exerted by chemotherapeutics promotes the Darwinian “survival of the fittest” cells. Drug-resistant cell sub-populations, incl. the “cancer stem cells” (CSCs; [5,6,9,10,32,33,34,35,36]), can pre-exist in the heterogeneous populations of prostate cancer cells [37,38,39]. Alternatively, adaptive phenotypic shifts, for instance the incomplete (type III) EMT-related transitions, favor the microevolution and selective expansion of the drug-resistant cells during prostate cancer chemotherapy [40]. To imitate this process in vitro, we used the approach based on the long-term “adaptive” exposition of prostate cancer DU145 and PC3 cells to chemotherapeutics. It resulted in the establishment of cell lineages that display high resistance to DCX and MTX. Notwithstanding the mechanism underlying their microevolution under DCX stress, FF interferes with the drug-resistance of prostate cancer cells as illustrated by additive attenuating effects of combined DCX/FF treatment on their expansion. This effect is executed by the induction of “suicidal” autophagy in DCX/FF-treated cells, which is followed by their apoptosis. It stays in concordance with the previous reports on the cytostatic FF activity [18]. Concomitantly, we have showed a long-term interference of the combined DCX/FF treatment with the displacement and diapedesis efficiency of drug-resistant cells. All these processes govern the selective recruitment of drug-resistant cancer cells to the invasive tumor fronts in vivo and their malignancy [19,41]. Thus, our current data are perhaps the first to directly show that FF can be used in the metronomic approaches of drug-resistant prostate tumors’ treatment. 

In our hands, the activation of P-gp (ABCB1) transporter accounted for a stable DCX/MDX-resistance of DU145 and PC3 cells that have undergone long-term DCX treatment. The efficiency of this system is illustrated by the relatively high viability and proliferation rate of DCX/MTX-treated DU145_DCX20/50 cells and by their sensitivity to the combined DCX/elacridar treatment. The attenuation of calcein efflux, observed in drug-resistant cells upon FF administration, demonstrates the interference of FF with ATP/P-gp-dependent DCX efflux system. The interference of FF with P-gp function has previously been reported in naïve tumor cells [42]. Apparently, it results in an intensified influx of DCX to DCX/FF-treated DU145 and PC3 cells, as confirmed by the disruption of microtubules and the induction of prostate cancer cell polyploidy. Thus, we show that FF interferes with drug-resistance, which has been acquired during their adaptive, DCX stress-induced microevolution. Similar cytostatic effects of FF on drug-resistant prostate cancer cells were observed in the presence of MTX. These FF activities were insensitive to chemical PPARα inhibition and ROS scavenging. It demonstrates that PPARα-/ROS-dependent signaling systems [24,25,43,44,45,46,47,48] are not involved in cell reactions to FF, even though the inhibition of DU145 proliferation by PPARα inhibition shows the significance of PPARα for the welfare of prostate cancer cells. We also ruled out the involvement of Cx43-mediated “by-stander effects” [49], because the inhibition of residual GJIC was observed in DCX/FF-treated DU145 populations, whereas PC3 cells are connexin-negative [38]. In turn, we observed lower ATP levels in DCX/FF-treated cells than in their DCX/elacridar-treated counterparts. This demonstrates that FF evokes energy deficit in prostate cancer cells. A corresponding effect has previously been described in glioblastoma cells, where FF impaired mitochondrial respiration at the level of complex I of the electron transport chain [50].

Until now, the research on the additive cytostatic effects of FF and chemotherapeutics was focused on the analysis of the synergy of their effects on naïve cancer cells. We are the first to comprehensively address the consequences and mechanisms of FF interference with acquired drug-resistance of prostate cancer cells. Collectively, we show a PPARα/ROS-independent interference of FF with the energy production in DCX-resistant cells, which leads to the inhibition of ATP/P-gp-dependent efflux system(s). Consequently, DCX, MTX and, potentially, other chemotherapeutics are accumulated inside the cells. Their interference with microtubular dynamics, with the intracellular P-gp transport (DCX, paclitaxel) or with P-gp synthesis (MTX) can lead to the further impairment of P-gp function and the intensification of DCX/MTX influx. Apparently, this vicious cycle results in the intoxication, mitotic catastrophe, and apoptosis of drug-resistant cells. The interference of FF with the energy production may also have profound consequences for the microevolution of drug-resistant prostate tumors. Actually, the cells that had survived the permanent DCX/FF-induced stress gave the offspring (DU145_DCX/FF lineage) that displayed relatively high DCX/FF sensitivity and a benign phenotype. A correlation between the resistance of DU145 and PC3 cells to DCX and MTX, and their sensitivity to FF suggests that oxidative respiration is intensified in drug-resistant DU145/PC3 lineages [51]. It may well be that DCX- and DCX/FF-induced metabolic reprogramming increases ATP supply in cancer cells to ascertain the efficient action of efflux pumps [50]. However, metabolic reprogramming can also be an Achilles heel of drug-resistant cancer cells, because it sensitizes drug-resistant cells to FF-induced imbalance between ATP demand and supply. Its precise mechanism(s) and clinical consequences require further study. However, these observations may illustrate a novel mechanism that underlies the interference of FF with the microevolution of drug-resistant prostate cancers, which potentially leads to the remission of drug-resistant tumors in vivo.

Further studies are also necessary to estimate the involvement of other drug-resistance mechanisms in cell reactions to DCX/FF-induced stress. In fact, the activation of FF-insensitive self-defense systems in DCX/FF-treated cells is suggested by their relatively weak apoptotic response to the combined DCX/FF treatment. “Suicidal” autophagy in DCX/FF-treated cells was followed by its “protective” variant in scarce cell sub-populations, which have survived the long-term DCX/FF treatment. FF may interfere with this process [52,53]; however, autophagy can confer survival advantage to scarce sub-populations of DCX/FF-treated drug-resistant cancer cells. Furthermore, the presence of CD133^+^/CD44^+^ CSC-like cells within the populations of drug-resistant cells (data not shown) was accompanied by a relatively high fraction of polyploid, polymorphonuclear “giant” cells in their DCX/FF-treated variants [54]. It indicates that these cells may account for the clonal expansion of CSCs [55,56] that underlies the microevolution of prostate cancer cell lineages under DCX/FF stress. 

Drug-resistance of cancer cells is a major challenge of current oncology. Therapeutic approaches of palliative prostate cancer treatment should be systemically tolerable and efficient against drug-resistant cancers. They should also interfere with the invasive front of cancer and with the microevolution of drug-resistant cells. Currently available chemotherapeutic strategies provide some symptom relief but are incapable of effectively interfering with the natural history of hormone-refractory prostate cancer. Usually, drug-resistance is developed in prostate tumors subjected to chemotherapy-related stress, following their castration-resistance. Concomitantly, chemotherapeutics evoke a number of adverse effects, which limit their long-term application and often enforce premature cessation of palliative chemotherapy, leading to inevitable and lethal relapses. Metronomic strategy based on the combined application of cytostatics (DCX or MTX) and fenofibrate can interfere with the drug-resistance of prostate cancer cells in vivo, thus reducing the effective doses of chemotherapeutics. It can also delay the metastatic cascade of prostate cancers. Furthermore, it can delay chemotherapy-induced microevolution, expansion and systemic dissipation of drug-resistant, invasive sub-clones of prostate cancer cells. Last but not least, FF has previously been shown to interfere with tumor angiogenesis and to augment endothelial barrier function [38,57,58]. Therefore, FF is a promising metronomic agent that can serve to enhance the efficiency of the palliative chemotherapy of prostate cancer. On the other hand, we have also detected the signs of DCX/FF resistance of prostate cancer cells. It may well be that prostate cancer cells can activate FF-insensitive, drug-resistance mechanisms that protect them against DCX/FF-induced stress in vivo. Such mechanisms include drug efflux-independent systems based on autophagy and/or on the function of polyploid “giant” cells, which may participate in tumor recurrence after the cessation of chemotherapy. Furthermore, it remains to be determined if FF can interfere with the drug-resistance of other tumors. The data from in vitro studies cannot be directly interpreted in terms of the clinical application of tested agents. Therefore, all these assays should also be performed on the cells from clinical samples and should be accompanied by epidemiologic studies on the interference of FF with prostate cancer progression. Nevertheless our data, in conjunction with the relatively high systemic tolerance of fenofibrate, suggest that FF can help to overcome basic limitations of chemotherapy in the treatment of elderly prostate cancer patients [29]. Thus, they add to the wide spectrum of cytostatic and anti-invasive activities of this FDA-approved drug [17,59]. 

## 4. Materials and Methods

### 4.1. Cell Cultures

Human prostate carcinoma DU145 (ATCC; HTB-81), PC3 cells (ECCAC; HTB-81) and their drug-resistant sub-lineages were routinely cultivated in DMEM/F12 HAM (Sigma, St. Louis, MO) medium supplemented with 10% fetal bovine serum (FBS) and antibiotics [19,38]. Human umbilical vein endothelial cells (HUVEC; Life Technologies Corporation, Carlsband, CA, USA) were cultured (up to six passages) in endothelial basal medium (EBM; Lonza, Basel, Switzerland) supplemented with 10% fetal bovine serum (FBS) and supplement cocktail (hydrocortisone, recombinant hEGF, bovine brain extract, gentamicin, amphotericin-B; all from Lonza [58]). For endpoint experiments, media supplemented with DCX and/or FF were added to cancer cell cultures at the concentrations given in the text. Culture media were supplemented with: docetaxel (DCX; 0.125–50 nM), mitoxantrone (MTX; 62.5–2000 nM) and/or fenofibrate (FF; 5–25 μM; F6020, Sigma, Saint Louis, MO, USA), GW6471 (10 μM; G5045), N-acetyl-L-cysteine (NAC; 1 mM; A9165, Sigma), elacridar (100 nM); sulfinpyranoze (500 μM), Fumitremorgin C (10 μM), 3-methyladenin (3MA; 0.5 μM; all from Sigma) at the time points indicated in the text. Chemical inhibitors were administrated at the time points indicated in the text and at the concentrations that secure their specific action and the lack of cytotoxic effects.

### 4.2. Establishment of DCX-Resistant DU145 Sub-Lineages

Prior to a pre-selection procedure, dose-response assay was performed to estimate the range of DCX concentrations that significantly inhibits the growth of naïve DCX145 and PC3 cells over a 3-day treatment. Because the treatment of DU145 cells with 1 nM DCX resulted in ca. 30% growth retardation, a selection strategy of cell exposure to DCX was initiated with the media supplemented with 1 nM DCX. Cells were seeded at the density of 2 × 10^4^ cells/cm^2^ into 6-well plates. The media were replenished after 3 days with the fresh medium containing 30% of DU145-conditioned medium to allow the cells to recover from chemotherapeutic stress, and the cells were cultivated in its presence for 3 days before a new portion of 1 nM DCX-containing medium was administered. After 3 cycles of therapy/recovery, the procedure was repeated in the presence of higher DCX concentration (Figure S3). To establish DU145_DCX20 cell line, DCX was sequentially applied at the concentrations of 1, 2, 5, 10, 20 nM. A corresponding procedure was employed to establish PC3_DCX20 cells. Additionally, DU145_DCX20 cells were subjected to 3 cycles of 50 nM DCX treatment to establish DU145_DCX50 cells. Alternatively, DU145 were consecutively cultivated in the presence of increasing DCX/FF concentrations to establish “super-resistant” DU145_DCX/FF sub-lineage. The following DCX/FF concentrations (nM/µM) were sequentially applied: 1/2.5, 2/5, 5/10, 10/20, 20/25. Since the continuous cell culture can result in alterations in cellular characteristics (including drug-resistance), untreated parental cells were cultivated alongside the treated cells as a control. The established subsets and sub-clones were cultivated in standard medium for ca. 15 generation times (five passages at 1:8). Stability of acquired resistance was assessed after freezing, thawing and following drug withdrawal throughout this period.

### 4.3. Cell Motility and Transmigration Assays

The movement of cancer cells was recorded using time-lapse Leica DMI6000B videomicroscopy system equipped with a temperature chamber (37 °C ± 0.2 °C)/(5% CO_2_), interference modulation contrast (IMC) optics and a cooled, digital DFC360FX CCD camera. DU145 and PC3 cells were seeded into 12-well plates at a density of 5000 (short-term incubation variant) or 1000 cells/cm² (long-term incubation variant). The cell trajectories were constructed from a sequence of cell centroid positions recorded for 6 h at 300 s time intervals (using a dry ×10, NA-0.75 objective) to estimate: total length of single cell trajectory (μm), speed of cell movement (i.e., total length of single cell trajectory/time of registration (μm/min) and total length of single cell displacement (Displacement; μm). These data were pooled and analyzed to calculate the averaged values of these parameters at the population level and to perform their statistical analysis (from no less than three independent experiments; number of cells >50 [60]). For transmigration assays, human umbilical vein endothelial cells (HUVEC) were seeded on coverslips at 2 × 10^4^ cells/well and grown to confluence for 72 h. Thereafter, CellTracer Orange CMRA (10 μM, Life Technologies)-stained cancer cells were seeded (1300/cm^2^) on HUVEC monolayers and incubated for 1 and 6 h [58]. Then, the specimens were stained against F-actin/DNA for microscopic estimation of the percentage of cancer cells capable of disrupting the endothelial continuum (transmigration). Transmigration of at least 200 cancer cells was analyzed for each group. 

### 4.4. Calcein Transfer Assay

Donor and acceptor cells were incubated in the presence of 2.5 nM DCX/25 μM FF for 24 h. Donor cells were stained with calcein/DiI (Life Technologies; C3099; 5 μM/10 μM) as described previously [61] and seeded onto the monolayers of acceptor cells at 1:50 ratio. After 1 h, a dye transfer from at least 200 donor cells per coverslip was analyzed using a Leica DMI6000B inverted fluorescence microscope (Leica; excitation—BP 470/40 nm; emission—BP 525/50) equipped with LasX software. It was further quantified as the percentage of donor cells, which successfully coupled with acceptor monolayer (coupling index; c_i_) and averaged number of coupled acceptor cells/a donor cell (coupling ratio; c_r_). Alternatively, co-cultures of donor and acceptor cells were trypsinized and fluorescence intensity of suspended cells was measured with a fluorescence-activated cell sorting (FACS) Aria system (Becton–Dickinson, Heidelberg, Germany; excitation wavelength of 488 nm). The emission of calcein and DiI was determined after filtering with a 530/30-nm (FL1) and a 575/26-nm bandpass filter (FL2), respectively. Each sample was measured three times at a total of 10 000–50 000 cells. GJIC was further quantified as the ratio of calcein stained acceptor to donor cells (coupling ratio). 3 independent experiments (N = 3) were performed for each experimental condition.

### 4.5. Immunofluorescence

Immunostaining of α-tubulin and vinculin was performed on formaldehyde/Triton X-100 fixed/permeabilized cells (FA; 3.7%; 20 min. in RT)/Triton X-100 (0.1%; 10 min. in RT). Intracellular Cx43 and P-gp localization was visualized in the cells fixed with methanol:acetone (7:3, −20 °C) for 15 min. After the incubation in the presence of 3% bovine serum albumin (BSA), primary antibodies: rabbit anti-Cx43 IgG (No. C6219, Sigma), mouse anti-vinculin IgG (No. V9131, Sigma), mouse anti-α-tubulin IgG and mouse anti-P-gp IgG (all from Sigma) were applied for 1 h. Then, the cells were labeled with Alexa 488-conjugated goat anti-mouse IgG (No. A11001, Invitrogen, Carlsbad, CA, USA), Alexa 488-conjugated goat anti-rabbit IgG (A11008, Invitrogen). Where indicated, the cells were counterstained with Alexa488 or TRITC-conjugated phalloidin (No. 49409 and 77418, Sigma) and Hoechst 33258 (No. B2883, Sigma) or 7AAD (No 51-2359KC, BD Pharminogen, San Diego, CA, USA) [38]. Image acquisition was performed with a Leica DMI6000B microscope (DMI7000 version; Leica Microsystems, Wetzlar, Germany) equipped with the total internal reflection fluorescence (TIRF) and differential interference contrast (DIC) and integrated modulation contrast (IMC) modules. Images were registered with 40×, NA-1.47 oil immersion objective in 37 °C/5%CO_2_ using 14-bit Hamamatsu 9100-02 EM-CCD camera controlled by the Leica Application Suite Advanced Fluorescence software. LAS-AF deconvolution software was used for image processing [39].

### 4.6. Proliferation, Cell Cycle and Apoptosis Assays 

Cells were seeded in triplicates into 24-well plates (Corning) at the density of 5 × 10^3^ cells/cm^2^, cultivated in the culture medium for 24 h, before the addition of the media containing DCX/FF and/or the inhibitors. After 48 h of cultivation, the cells were harvested and re-suspended in the original culture medium. Counting was performed with a Coulter Z2 Counter (Beckman Coulter Inc., Fullerton, CA, USA). Doubling times (DT) were calculated according to the formula: DT=Tln2/ln(X_e_/X_b_), where: T is the incubation time; X_b_ and X_e_ are the cell numbers at the beginning and at the end of the incubation time, respectively. 

For DNA content (cell-cycle) analyses, the cells were seeded into 6-well plates (Corning) at the density of 5×10^3^ cells/cm^2^, cultivated in culture medium for 24 h. Thereafter, media containing FF (25 μM) and/or DCX (2.5 nM) were administered for 48 h, followed by their collection, trypsinization of the attached cells, and their resuspension in the original medium, fixation with EtOH (70% at −20 °C) and propidium iodide staining (PI; 50 µg/mL) in the presence of RNAseA (1 mg/mL). FlowSight® imaging cytometer (Amnis Corp., Seattle, WA, USA) was employed for the analyses of DNA content. At least 3x10^5^ cells were analyzed for each condition. Concomitantly, the attached cells were fixed with EtOH (70% at −20 °C), stained with Hoechst33258 (Sigma, 1 µg/mL) and the morphology of their nuclei was analyzed with fluorescence microscopy. For the analyses of cell apoptosis, trypsinized cells were re-suspended in original medium and subjected to AnnexinV/propidium iodide staining according to the manufacturer’s protocol (BD Pharmigen). Flow cytometric detection of apoptotic cells was performed with a FACSAria system (Becton–Dickinson, Heidelberg, Germany [62]. At least 5x10^4^ cells were analyzed for each condition.

### 4.7. Intracellular Adenosine Triphosphate (ATP) Assay

Intracellular ATP contents were estimated using luminescence ATPlite detection assay system (cat. no. 6016947; Perkin-Elmer, Warszawa, Poland) according to the manufacturer’s protocol. Briefly, the cells were cultivated in the presence of DCX/FF/elacridar for 48 h, lysed and the equal volumes of cell lysates were transferred to white plates for collection of luminescence signals using the Infinite M200 reader. ATP contents per population/cell were estimated from calibration curves to estimate the effect of the agents on cell viability and on the efficiency of ATP production at the single cell and population level. 

### 4.8. Analyses of DCX-Resistance of DU145 Cells in Vivo

Severe combined immunodeficient (SCID) mice of ages 5 weeks were purchased from Charles River Laboratories and maintained in a temperature-controlled, pathogen-free room. Before the experiments, the animals were quarantined and acclimatized for two weeks. Mice were kept in community cages on a standard laboratory diet with free access to drinking water and a 12 h day/night regime. All animals were handled according to the approved protocols and guidelines of 2^nd^ Local Ethics Committee for Experiments on Animals at the Jagiellonian University in Cracow (Dec No. 290/2017). Cancer cells were mixed with BD Matrigel basement membrane matrix high concentration (1:1 in PBS; BD Biosciences), and 40 μL of the cold liquid Matrigel-mixed (1.5 × 10^5^) cells was slowly injected subcutaneously into abdominal flank of SCID mice. The mice were observed for 3 to 5 weeks for the appearance and development of tumors. Every 4 days, docetaxel (20 mg/kg) was administrated intraperitoneally to the mice. The tumor volume was calculated according to the following formula: V = (Π/6)a×b×c, where a, b, c are perpendicular diameters of the ellipsoid approximating the shape of the tumor. 

### 4.9. Tandem Mass Spectrometry 

Tandem mass spectrometry (liquid chromatography–mass spectrometry, LC–MS/MS) was performed with the reversed-phase liquid chromatography (RPLC) system (UltiMate 3000 RSLCnano System, Thermo Fisher Scientific, Waltham, MA USA) coupled Q-Exactive mass spectrometer (Thermo Scientific); mass spectrometer. Lysates of DU145 cells and their DCX-resistant counterparts cells were prepared for shotgun LC–MS/MS measurements using the filter assisted sample preparation (FASP) method. The peptide samples were loaded onto a trap column (Acclaim PepMap 100 C18, Thermo Fisher Scientific) and further separated on analytical column (Acclaim PepMap RSLC C18, Thermo Fisher Scientific). Eluting peptides were ionised using a Digital PicoView 550 nanospray source (New Objective) and acquired in a MS data dependent mode using top twelve method. The LC–MS/MS data were analyzed using Proteome Discoverer 1.4 and a MASCOT server against the Swissprot_201802 database. Search result validation was performed using the Percolator algorithm.

### 4.10. Calcein Efflux Assay

For the calcein-AM efflux assay, cells were detached by trypsin/EDTA treatment, re-suspended in 0.25 μM calcein-AM solution in PBS and incubated for 30 min. at 37 °C. Then, the cells were washed and splitted to quantify P-gp activity by fluorescence microscopy or flow cytometry. Flow cytometric detection of calcein fluorescence was performed with BD LSR Fortessa X-20 flow cytometer, using 488 nm excitation and a 525 nm band pass filter. For each analysis, 10,000 events were recorded, according to a particle diameter exceeding 8 μm. For the fluorimetric analyses of calcein-stained specimens, the stacks of fluorescence images of at least 16 randomly chosen confluent culture regions were collected in the green channel (A4; excitation—BP360/40 nm; emission—BP470/40 nm). In each experiment, the stacks were obtained with the same excitation/exposure settings (excitation/camera gain/time of exposition). The fluorescence index was estimated for each stack with LasX software (Leica) and calculated for each specimen [63]. 

### 4.11. Analyses of Autophagy

Cells were seeded into 12-well plates (Corning) at the density of 5 × 10^3^ cells/cm^2^ and cultivated in culture medium for 24 h, before the administration of the media containing FF (25 µM) and/or DCX (2.5 nM) for 48 h. After the incubation, the cells were treated with an Autophagy Assay Kit (MAK138, Sigma) according to the manufacturer’s protocol. Fluorescence intensity was measured at 360/520 nm wavelengths (excitation/emission respectively) with 40×, NA-1.47 oil immersion objective in 37 °C/5%CO_2_ and 14-bit Hamamatsu 9100-02 EM-CCD camera controlled by the Leica Application Suite Advanced Fluorescence software. 

### 4.12. Statistical Analysis

All data were expressed as mean ± SEM from at least three independent experiments (N = 3). The statistical significance was tested with t-Student and Mann-Whitney tests. Statistical significance was shown at *^,#^ *p* ≤ 0.05.

## 5. Conclusions

Drug-resistance is a major obstacle in prostate cancer treatment. Collectively, our data show that fenofibrate sensitizes drug-resistant prostate cancer cells to chemotherapeutics via the inactivation of their ATP-dependent drug-efflux systems. This effect conceivably results from the interference of fenofibrate with the energy metabolism of cancer cells and does not induce the microevolution of their fenofibrate-resistant lineages. This novel PPARα/ROS-independent mechanism of fenofibrate cytotoxicity opens perspectives for elaboration of new metronomic strategies of prostate cancer treatment that would target the energy metabolism/drug-resistance of cancer cells. 

## Supplementary Materials

The following are available online at http://www.mdpi.com/2072-6694/11/1/77/s1: Figure S1: The effect of DCX and/or FF on GJIC and proliferation of DU145 cells; Figure S2: Inhibitory effect of FF on the multi-drug resistance of PC3 cells; Figure S3: Experimental protocol for the establishment of DCX-resistant DU145 and PC3 sub-lineages; Figure S4: Phenotype and drug-resistance of DU145_DCX20 and DU145_DCX50 cells; Figure S5: Effect of FF on drug-resistance of DU145_DCX20 and DU145_DCX50 cells; Figure S6. Long-term sensitivity of drug-resistant DU145 cells to DCX and FF; Figure S7: Effect of FF on the activity of MDR transporters in DCX-resistant DU145 cells; Figure S8: Reactivity of DU145_DCX/FF cells to combined DCX and FF.
