# Supplementary material for: Fenofibrate Augments the Sensitivity of Drug-Resistant Prostate Cancer Cells to Docetaxel
Source: Cancers (Basel). 2019 Jan 11;11(1):77. doi: 10.3390/cancers11010077 (PMC6356694; doi:10.3390/cancers11010077)
Supplement: Supplementary file 1 [file cancers-11-00077-s001.pdf]

Supplementary Material

Fenofibrate Augments the Sensitivity of Drug-Resistant Prostate Cancer Cells to Docetaxel

Marcin Luty, Katarzyna Piwowarczyk, Anna Łabędź-Masłowska, Tomasz Wróbel, Małgorzata Szczygieł, Jessica Catapano, Grażyna Drabik, Damian Ryszawy, Sylwia Kędracka-Krok, Zbigniew Madeja, Maciej Siedlar, Martyna Elas and Jarosław Czyż

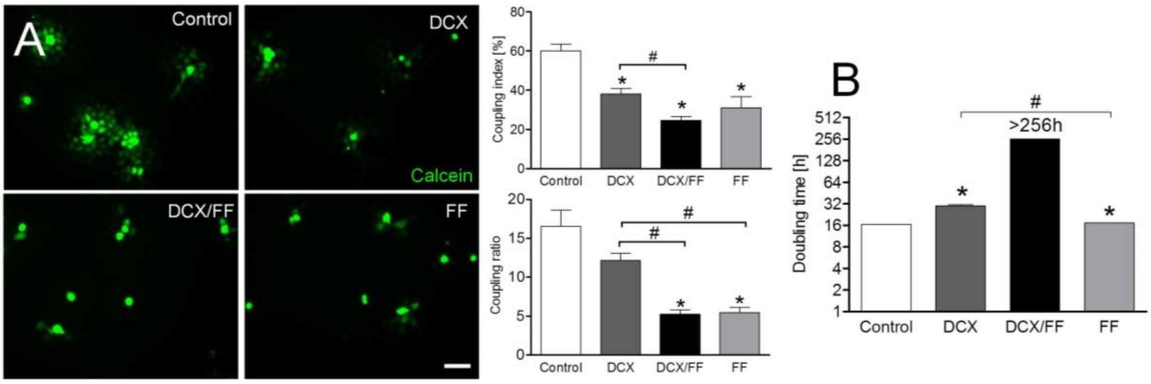

Figure S1. The effect of DCX and/or FF on GJIC and proliferation of DU145 cells. (**A**) DU145 cells were cultivated as in Figure 1A for 24 h and gap-junctional intercellular coupling (GJIC) was estimated by calcein transfer assay. Coupling index shows the % of donor cells coupled with at least 1 acceptor cell, whereas Coupling ratio shows the averaged number of coupled (calcein^{+}) recipient cells/donor cell calculated with FACS. At least 50 000 events, classified based on their bright field ratios were analyzed. (**B**) DU145 cells were cultivated in the presence of DCX (2.5 nM) and/or FF (25 μM) and their doubling times were estimated after 48 hours. Error bars represent SEM. Data are representative of at least three independent experiments (N > 3). Statistical analyses were performed with the t-Student test (* $p \leq 0.05$ vs. untreated cells or # $p \leq 0.05$ vs. the control indicated by the brackets). Scale bar: 100 μm. Note that FF sensitizes DU145 cells to DCX.

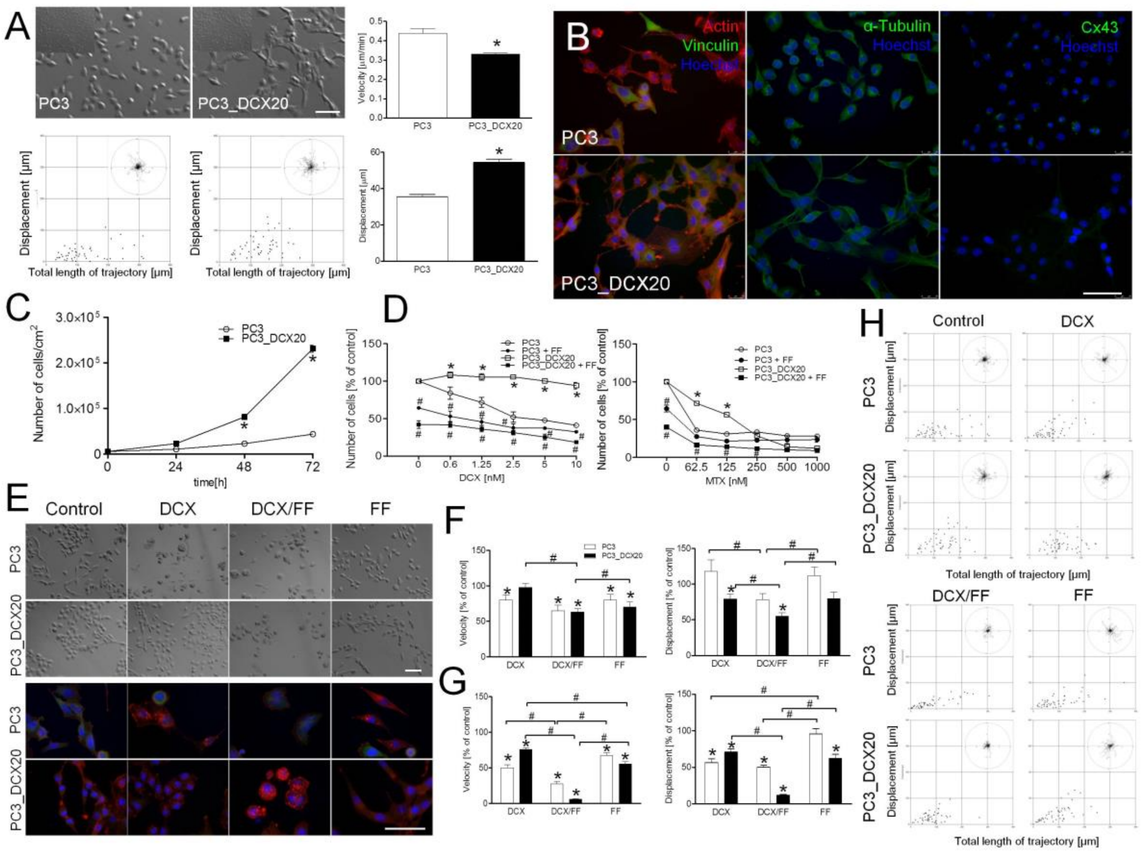

Figure S2. Inhibitory effect of FF on the multi-drug resistance of PC3 cells. (**A**) Drug-resistant PC3_DCX20 cells are characterized by a slightly higher invasive potential than their naïve PC3 counterparts, as illustrated by the increased efficiency of their displacement in control conditions (* $p \leq 0.05$ vs. PC3 cells tested with non-parametric Mann-Whitney test). Concomitantly, these cells display fibroblastoid morphology, which is correlated with the formation of more abundant stress fibers in the absence of any distinct differences in the architecture of microtubules and Cx43 levels (**B**). PC3 cells were also characterized by a relatively high proliferation rate (**C**) and a very low sensitivity to DCX/mitoxantrone (MTX) (**D**; * $p \leq 0.05$ vs. PC3 cells and # $p \leq 0.05$ vs. PC3_DCX20 cells tested with T-Student test). Cell proliferation was estimated 48 h after the administration of agents. Fenofibrate (25 μM) considerably increased the cytostatic effects of DCX/MTX on PC3_DCX20 cells. It also exerted a more prominent inhibitory effect on the proliferation of PC3_DCX20 cell in the absence of DCX/MTX. These effects were correlated with the changes in cell morphology, especially with the loss of rear-front polarization (**E**; red—actin; blue—DNA)**,** and with the inhibition of cell motility immediately after the administration of the drugs (**F**) and after 48 h treatment (**G**; * $p \leq 0.05$ vs. non-treated PC3 cells (100% control) or # $p \leq 0.05$ vs. the control indicated by brackets; tested with non-parametric Mann-Whitney test), which was even more prominent than that of naïve PC3 cells. (**H**) Cell trajectories depicted as circular diagrams (axis scale in μm) drawn with the initial point of each trajectory placed at the origin of the plot (registered for 6 h; N > 50). Error bars represent SEM. Scale bars: 50 μm (**A**, **B**) and 100 μm (**E**).

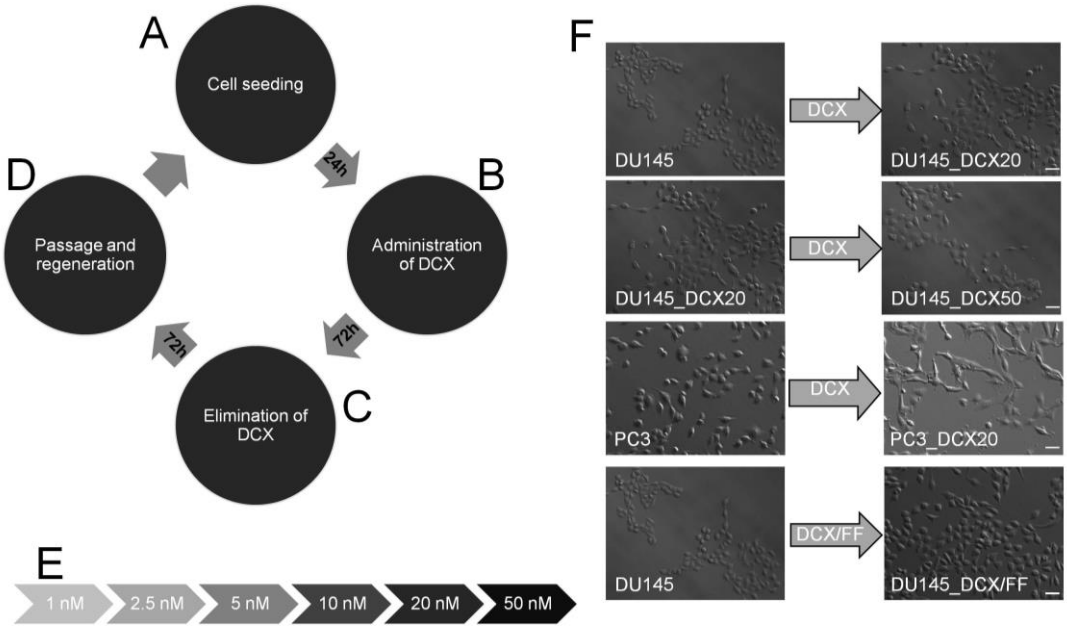

Figure S3. Experimental protocol for the establishment of DCX-resistant DU145 and PC3 sub-lineages. Cells were seeded at the density of 2×10^4 cells/cm^2 into 6-well plates, cultured for 24 h (**A**) and the medium supplemented with 1 nM DCX was added to the cultures for the next 72 h (**B**). Then, the media were replenished with the fresh medium containing 30% of DU145-conditioned medium for 3 days (**C**) before a new portion of 1 nM DCX-containing medium was administered (**B**)**.** After 3 cycles of therapy/recovery, the cells were passaged (**D**) and the procedure was repeated in the presence of higher DCX concentration (**D**). To establish DU145_DCX20 cell line DCX was sequentially applied at the concentrations of 1, 2.5, 5, 10, 20 nM. A corresponding procedure was employed to establish PC3_DCX20 cells. Additionally, DU145_DCX20 cells were subjected to 3 cycles of 50 nM DCX treatment to establish DU145_DCX50 cells (**E**). Alternatively, DU145 were consecutively cultivated in the presence of increasing DCX/FF concentrations to establish "super-resistant" DU145_DCX/FF sub-lineage (**F**).

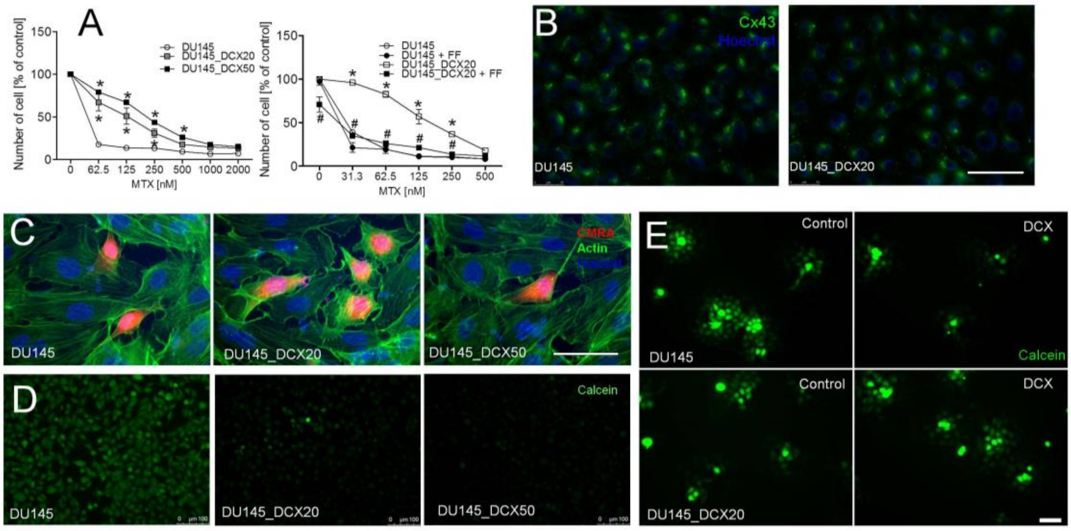

Figure S4. Phenotype and drug-resistance of DU145_20 and DU145_DCX50 cells. (**A**) DCX-resistant DU145 cells display relatively high resistance to MTX (left), whereas 25 µM FF considerably increases their sensitivity to this drug (right). Cells were cultivated in the presence of mitoxantrone (MTX; 31.25–2000 nM) and/or 25 µM FF and their proliferation was estimated after 48 h (* $p \leq 0.05$ vs. DU145 cells and # $p \leq 0.05$ vs. DU145_DCX20 cells tested with T-Student test). (**B**) Intracellular localization of Cx43 in naïve DU145 and DU145_DCX20 cells. (**C**) Trans-endothelial migration efficiency of DU145 and DCX-resistant DU145_DCX20 and DCX50 cells was estimated in actin/vinculin-stained specimens. Cancer cells were additionally stained with CMRA. (**D**) The activity of efflux pumps in DCX-resistant DU145 cells was estimated with calcein efflux assay in control conditions. (**E**) DU145 and DU145_DCX20 cells were cultivated (**A**) for 24 h and gap-junctional intercellular coupling (GJIC) in their populations was estimated by calcein transfer assay in the presence/absence of DCX. Scale bars: 50 (**B**, **C**) and 100 µm (**D**, **E**).

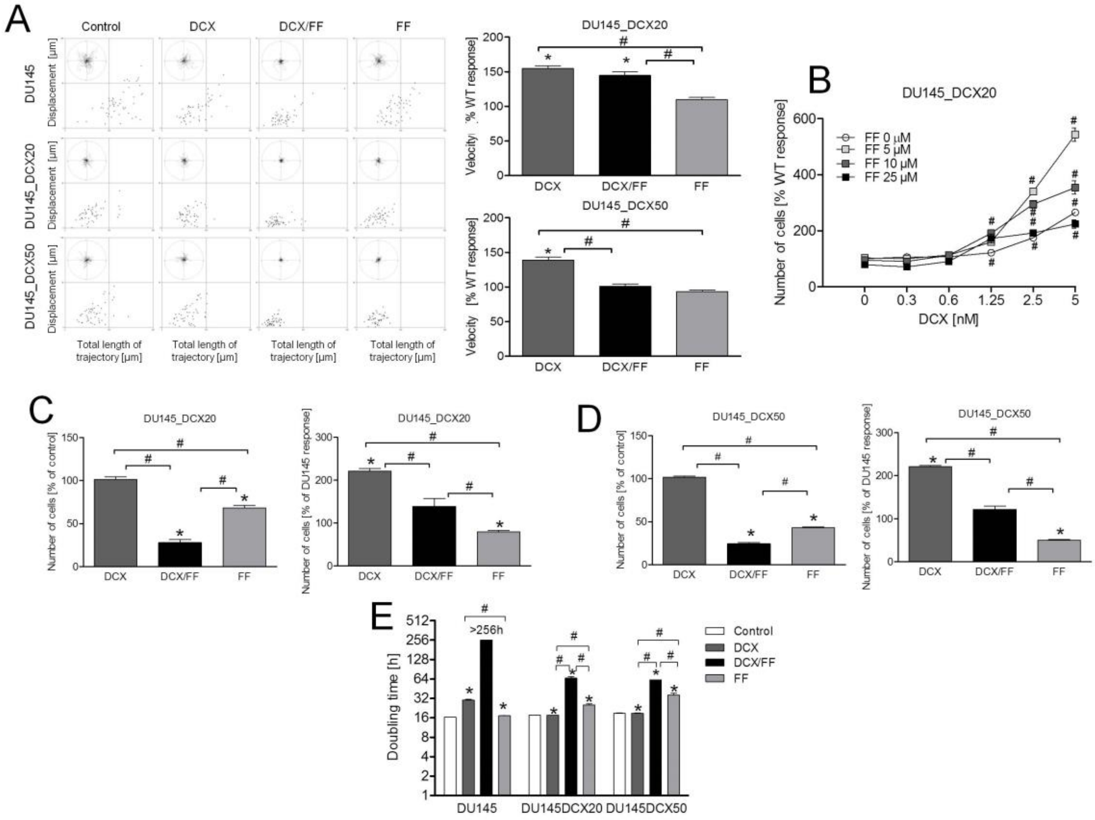

Figure S5. Effect of FF on drug-resistance of DU145_DCX20 and DU145_DCX50 cells. (**A**) Motility of DU145_DCX20 and DU145_DCX50 cells. Cell motility was estimated immediately after drug administration. Cell trajectories are depicted as circular diagrams (axis scale in µm) drawn with the initial point of each trajectory placed at the origin of the plot (registered for 6 h; N > 50). Dot-plots and column charts show movement parameters at the single cell and population level, respectively. Averaged velocity of DU145_DCX20 and DU145_DCX50 cell movement in the presence of 2.5 nM DCX/25 µM FF DCX was calculated in relation to the behavior of naïve DU145 cells in the corresponding conditions. Statistical significance was analyzed with non-parametric the Mann-Whitney test vs. the response of naïve DU145 cells to corresponding conditions (* $p \le 0.05$) or vs. the variant indicated by the bracket (# $p \le 0.05$). (**B**) Effect of DCX (0.3–5 nM) and FF (5–25 µM) on the proliferation of DU145_DCX20 cells calculated in relation to the behavior of naïve DU145 cells in the corresponding conditions. Statistical significance was analyzed with the t-Student test vs. the response of naïve DU145 cells to corresponding conditions (* $p \le 0.05$). (**C**,**D**) Proliferation of DU145_DCX20 (**C**), of DU145_DCX50 cells (**D**) in the presence of 2.5 nM DCX and/or 25 µM FF calculated as a % of control (left) or in relation to the behavior of naïve DU145 cells in the corresponding conditions (right). Statistical significance was analyzed with the t-Student test vs. non-treated naïve DU145 cell (left) or the response of naïve DU145 cells to corresponding conditions (right; * $p \le 0.05$) or vs. the variant indicated by the bracket (# $p \le 0.05$). (**E**) Doubling times of DU145_DCX20 and DU145_DCX50 cells cultivated in the presence of 2.5 nM DCX and/or 25 µM FF Statistical significance was analyzed with t-Student vs. non-treated naïve DU145 cells (* $p \le 0.05$) or vs. the variant indicated by the bracket (# $p \le 0.05$). Data representative of at least three independent experiments (N > 3). Error bars represent SEM. Note the additive effects of FF and DCX on the welfare and motility of DCX-resistant DU145 cells.

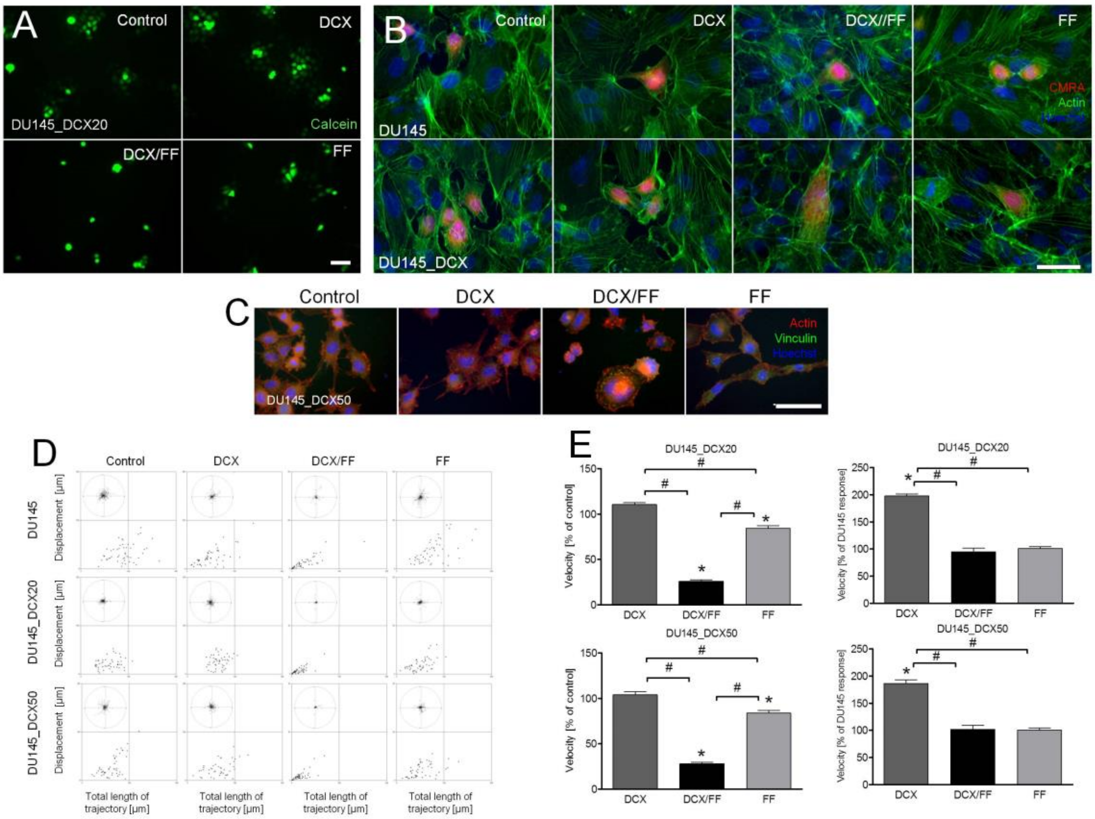

Figure S6. Long-term sensitivity of drug-resistant DU145 cells to DCX and FF. (**A**) DCX-resistant DU145 cells were incubated in the presence of 2.5 nM DCX and/or 25 µM FF for 24 h. GJIC was estimated by calcein transfer assay and calculated as Coupling index and Coupling ratio (cf. Figure 3). (**B**) Naïve DU145 and DU145_DCX20 cells were cultivated as in (**A**) and their transmigration through HUVEC continuum was visualized to estimate transendothelial migration assay (cf. Figure 3). (**C**) Actin cytoskeleton architecture of DU145_DCX50 cells cultured in the presence of 2.5 nM DCX/25 µM FF DCX. (**D**) DU145_DCX20 and DU145_DCX50 cell motility was estimated 48 h after the administration of the drugs. Cell trajectories are depicted as circular diagrams (axis scale in µm) drawn with the initial point of each trajectory placed at the origin of the plot (registered for 6 h; N>50). Dot-plots show movement parameters at the single cell level. (**E**) Averaged velocity of DU145_DCX20 and DU145_DCX50 cell movement in the presence of 2.5 nM DCX/25 µM FF DCX, calculated as a % of control or in relation to the behavior of naïve DU145 cells in the corresponding conditions. Data representative of at least three independent experiments (N > 3). Error bars represent SEM. Statistical significance was analyzed with non-parametric the Mann-Whitney test vs. non-treated control (**E** left; * $p \leq 0.05$), vs. the response of naïve DU145 cells to corresponding conditions (**E**; right; * $p \leq 0.05$) or vs. the variant indicated by the brackets (# $p \leq 0.05$). Scale bars: 50 µm. Note that FF strengthens the long-term effect of DCX on the invasive potential of DCX-resistant DU145 cells.

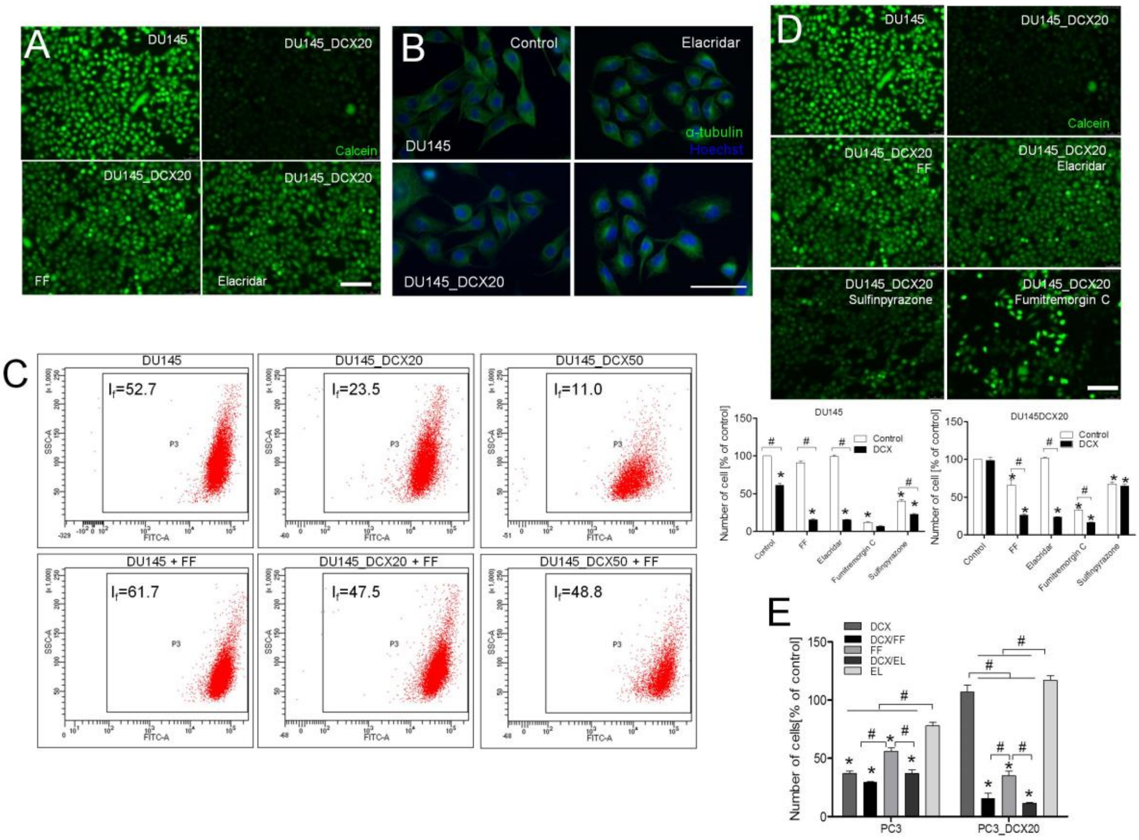

Figure S7. Effect of FF on the activity of MDR transporters in DCX-resistant DU145 cells. (**A**) DU145 and DU145_DCX20 cells were incubated in the presence of 2.5 nM DCX and the architecture of microtubules was visualized with fluorescence microscopy. (**B**) DU145_DCX20 cells were incubated in the presence of Elacridar or FF (25 µM) and the efficiency of the efflux systems was measured by calcein efflux assay. (**C**) Naïve DU145 and DU145_DCX20 cells were incubated in the presence of FF (25 µM) and the efficiency of the efflux systems was measured by FACS-assisted calcein efflux assay. (**D**) Chemical inhibition of ABCC1 (by Sulfinpyranoze) and ABCG2 (by fumitremorgin C) does not affect calcein efflux of DCX-treated DU145_DCX20 cells. DU145_DCX20 cells display lower sensitivity to sulfapyrazone than their DU145 counterparts, whereas their sensitivity to fumitremorgin C is similar. Statistical significance was analyzed with the t-Student test vs. non-treated DU145 (left), DU145_DCX20 control (right; * $p \leq 0.05$) or vs. the control indicated by the brackets (# $p \leq 0.01$). (**E**) PC3 and DCX-resistant PC3 cells were incubated in the presence of 2.5 nM DCX and the effect of elacridar (0.1 µM) and FF (25 µM) on their proliferation was estimated after 48 h of incubation. Statistical significance was analyzed with the t-Student test vs. non-treated PC3 control (* $p \leq 0.05$) or vs. the control indicated by the brackets (# $p \leq 0.01$). Error bars represent SEM. Scale bar: 100 µm. Note the sensitivity of drug-efflux systems in DU145 and PC3 cells and their proliferation to elacridar.

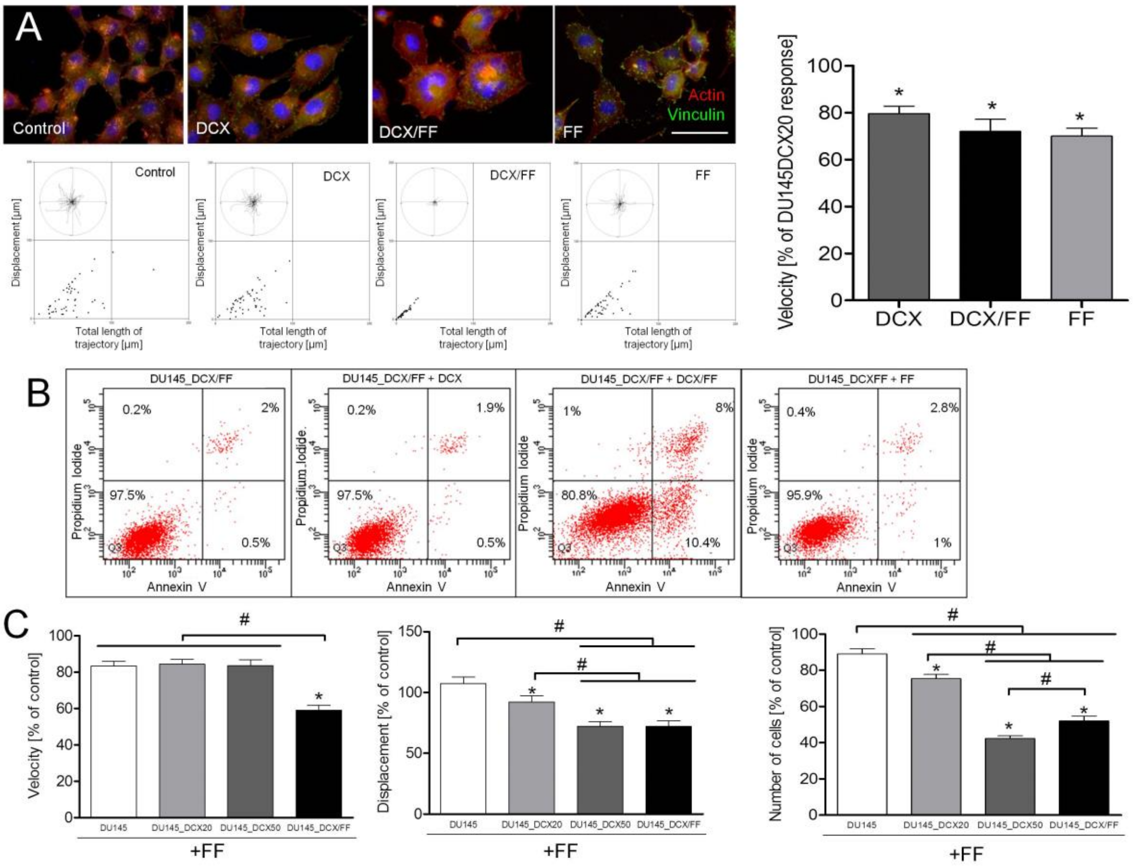

Figure S8. Reactivity of DU145_DCX/FF cells to combined DCX and FF. (**A**) The effect of 2.5 nM DCX/25 µM FF on the architecture of actin cytoskeleton and motility of DU145_DCX/FF cells, estimated by immunofluorescence and time-lapse videomicroscopy, respectively. Motility of DU145_DCX/FF cells cultivated in the presence of DCX (2.5 nM) and/or FF (25 µM) for 48 hours is calculated as a % of DU145_DCX20 control. Cell trajectories are depicted as circular diagrams (axis scale in µm) drawn with the initial point of each trajectory placed at the origin of the plot (registered for 6 h; N > 50). Dot-plots and column charts show movement parameters at the single cell and population level, respectively. Statistical significance was analyzed with the nonparametric Mann-Whitney test vs. the response of naïve DU145 cells to corresponding conditions (* $p \leq 0.05$). (**B**) Pro-apoptotic effects of the combined DCX/FF treatment in the populations of DU145_DCX/FF cells visualized with AnnexinV/PI test. Compensated dot-plots comprise 50 000 events. (**C**) Effect of FF on the proliferation of DU145 cells and their DCX-resistant counterparts, calculated in relation to their behavior in control conditions. Statistical significance was analyzed with the non-parametric Mann-Whitney test (left, middle), with t-Student test (right) vs. non-treated DU145 cells (right; * $p \leq 0.05$) or vs. the control indicated by the brackets (# $p \leq 0.05$). Error bars represent SEM. Scale bar: 50 µm.
